# Cornual Pregnancy After Ipsilateral Salpingectomy

**DOI:** 10.7759/cureus.17244

**Published:** 2021-08-17

**Authors:** Carol A Knight, Rachel E Bridwell, Brit Long, Sarah Goss

**Affiliations:** 1 Emergency Medicine, Brooke Army Medical Center, San Antonio, USA

**Keywords:** cornual pregnancy, interstitial pregnancy, ectopic pregnancy, post-salpingectomy, ipsilateral ectopic

## Abstract

Ectopic pregnancy is a serious diagnosis occurring in 1-2% of all pregnancies, causing significant morbidity and mortality if unrecognized. Management of ruptured ectopic pregnancy typically includes salpingectomy, which decreases the risk for repeat ectopic pregnancies. In rare cases after salpingectomy, non-viable implantation may occur on the ipsilateral side of prior surgery. We present a patient with a cornual pregnancy on the ipsilateral side of her prior ectopic pregnancy and salpingectomy.

## Introduction

An ectopic pregnancy is embryonic implantation in a location outside of the uterine cavity. The majority of ectopic pregnancies implant in the fallopian tube [1]. In cases of extensive tubal damage to include ectopic rupture or uncontrolled bleeding, salpingectomy is often the preferred approach to the management of a ruptured ectopic pregnancy [1]. In addition to hemodynamically stabilizing the patient and removing the non-viable pregnancy, salpingectomy decreases the incidence of repeat ectopic pregnancies by eliminating a venue for future extrauterine implantation [2]. However, implantation in or near the fallopian stump may occur in the setting of a subsequent ectopic pregnancy. Approximately 2-4% of ectopic pregnancies are cornual (often referred to as interstitial) pregnancies, or a pregnancy where the embryo implants in the junction between the fallopian tube and the uterus [3]. This predominantly non-viable form of ectopic pregnancy can result in increased morbidity and mortality due to myometrial distensibility and thus delayed diagnosis and increased risk of rupture [3]. We present a unique case of a cornual pregnancy on the ipsilateral side of a previous ectopic pregnancy status post salpingectomy.

## Case presentation

A 38-year-old gravida five para two at six weeks gestation based on last menstrual period presented to the emergency department (ED) for an episode of sharp abdominal pain in the left lower quadrant that occurred 10 hours prior to presentation without vaginal bleeding. The patient was concerned because her last pregnancy, which was terminated three months prior to presentation, resulted in a left-sided ectopic pregnancy, requiring a left salpingectomy. Of note, this current pregnancy was natural, unassisted by fertility treatments. On examination, her blood pressure was 119/72 mm Hg, heart rate 66 beats per minute, respiratory rate 18 breaths per minute, oxygen saturation 98% on room air, and oral temperature 98.0 degrees Fahrenheit. Her examination was notable for mild left lower abdominal tenderness without rebound or guarding. Laboratory assessment was notable for a beta-human chorionic gonadotropin of 14,448 mIU/mL and a normal complete blood count. 

**Figure 1:**
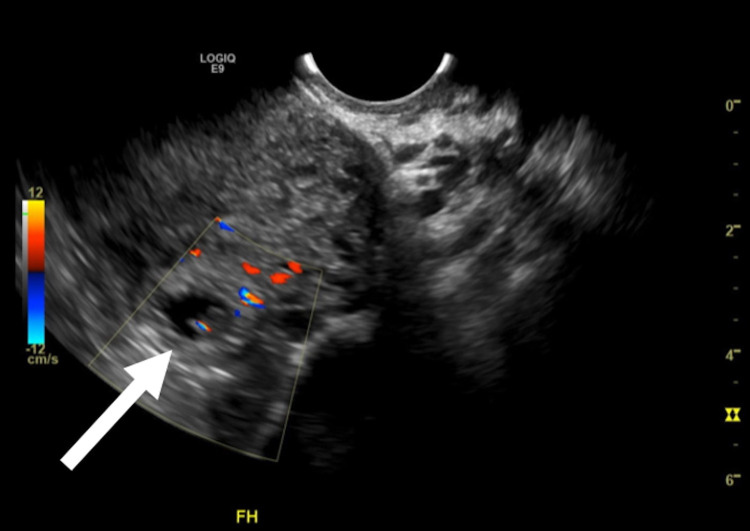
Six-week pregnancy on ultrasonography showing pregnancy (arrow) within the left fallopian tube stump.

A comprehensive transvaginal ultrasound revealed a fetus of six weeks and zero days by crown-rump length with a fetal heart rate of 142 beats per minute situated in the remnant (“stump”) of the left fallopian tube (Figure 1). Obstetrics was consulted for operative management, and the patient successfully underwent cornual resection of the identified cornual ectopy pregnancy. 

## Discussion

Ectopic pregnancy is defined as any pregnancy that implants outside of the uterine cavity, occurring in the fallopian tube in 95% of cases [4,5]. A cornual ectopic pregnancy is a pregnancy that implants in the upper and lateral uterine cavity [6].^ ^Interstitial pregnancy, or a pregnancy that implants within the proximal intramural portion of the fallopian tube, is often used synonymously in literature with cornual pregnancies due to their proximity in implantation. Both cornual and interstitial ectopic pregnancies comprise 2-4% of ectopic pregnancies.

History of prior ectopic pregnancy, pelvic inflammatory disease, or in vitro fertilization increases the risk of a cornual pregnancy [7]. Ipsilateral fallopian-implanted ectopic pregnancy in the setting of prior salpingectomy is extremely rare with few cases noted in the literature [8]. However, in a study of 32 cases of interstitial pregnancies, history of ipsilateral or bilateral salpingectomy was present in 37.5% of the patients [3]. Cornual and interstitial pregnancies can present with symptoms of a classic ectopic pregnancy such as vaginal bleeding and abdominal pain. However, since implantation occurs in close proximity to viable portions of the uterus, difficulty in ultrasound identification can delay diagnosis [6]. In addition, because the myometrium is significantly more distensible in cornual pregnancies than in fallopian ectopic pregnancies, cornual pregnancies can go undiagnosed up to 14 weeks [3]. Vascularity increases with gestational age, resulting in significant fetal and maternal hemorrhage in the setting of rupture. Mortality rate of cornual implantation is 15 times higher than the mortality of a tubal ectopic pregnancy, 2-2.5% and 0.14%, respectively, highlighting the importance of early identification in the ED [9].

Due to this risk of severe hemorrhage and mortality, emergency clinicians must consider this diagnosis in the setting of a patient with prior ipsilateral salpingectomy. With rapid identification, appropriate imaging modalities, and early obstetrical consultation, morbidity and mortality can be minimized for this population.

## Conclusions

Ectopic pregnancy is a common obstetrical emergency that presents to the ED and may result in significant morbidity and mortality. While most ectopic pregnancies implant in the fallopian tube, cornual and interstitial pregnancies pose a significant risk to the pregnant patient due to rupture that often occurs later in the first trimester. Rupture is associated with catastrophic hemorrhage, increasing mortality 15-fold in comparison to fallopian implantation. As demonstrated by this case, prior salpingectomy does not exclude the risk of non-viable implantation on the side of prior ectopic pregnancy. Although rare, ipsilateral ectopic pregnancies after salpingectomy can occur and must be considered in the evaluation of possible ectopic pregnancy.
